# Nocturnal Heart Rate Variability Might Help in Predicting Severe Obstructive Sleep-Disordered Breathing

**DOI:** 10.3390/biology12040533

**Published:** 2023-03-31

**Authors:** Rosario Statello, Stefano Rossi, Francesco Pisani, Matteo Bonzini, Roberta Andreoli, Agnese Martini, Monica Puligheddu, Pierluigi Cocco, Michele Miragoli

**Affiliations:** 1 Department of Medical Sciences and Public Health, University of Cagliari, 09124 Cagliari, Italy; 2 Department of Medicine and Surgery, University of Parma, 43121 Parma, Italy; 3 Child Neuropsychiatric Unit, Department of Human Neuroscience, Sapienza University of Rome, 00185 Rome, Italy; 4 Occupational Medicine, Fondazione IRCCS Ca’ Granda Ospedale Maggiore Policlinico, 20122 Milan, Italy; 5 Department of Clinical Sciences and Community Health, University of Milan, 20122 Milan, Italy; 6 CERT, Center of Excellence for Toxicological Research, University of Parma, 43121 Parma, Italy; 7 INAIL, The National Institute for Insurance against Accidents at Work, Department of Medicine, Epidemiology, Workplace and Environmental Hygiene, 00144 Rome, Italy; 8 Centre for Occupational and Environmental Health, Division of Population Health, Health Services Research & Primary Care, University of Manchester, Manchester M13 9PL, UK; 9 Department of Molecular Cardiology, Humanitas Research Hospital, IRCCS, 20089 Rozzano, Italy

**Keywords:** obstructive sleep apnea, heart rate variability, autonomic nervous system, breathing, sympathetic, vagal, diagnostic marker

## Abstract

**Simple Summary:**

Obstructive sleep apnea (OSA) is associated with behavioral, cardiovascular, and metabolic dysfunction. Autonomic changes have also been described in patients with OSA. In this study, we measured the heart rate variability (HRV) on electrocardiogram (ECG) tracings of OSA patients as a measure of cardiac autonomic regulation and putative predictor of disturbed breathing during nighttime. Our findings confirm an altered cardiac autonomic regulation in OSA patients during nighttime and identify a possible role of nighttime HRV in predicting sleep breathing disorders.

**Abstract:**

Obstructive sleep apnea (OSA) can have long-term cardiovascular and metabolic effects. The identification of OSA-related impairments would provide diagnostic and prognostic value. Heart rate variability (HRV) as a measure of cardiac autonomic regulation is a promising candidate marker of OSA and OSA-related conditions. We took advantage of the Physionet Apnea-ECG database for two purposes. First, we performed time- and frequency-domain analysis of nocturnal HRV on each recording of this database to evaluate the cardiac autonomic regulation in patients with nighttime sleep breathing disorders. Second, we conducted a logistic regression analysis (backward stepwise) to identify the HRV indices able to predict the apnea–hypopnea index (AHI) categories (i.e., “Severe OSA”, AHI ≥ 30; “Moderate-Mild OSA”, 5 ≥ AHI < 30; and “Normal”, AHI < 5). Compared to the “Normal”, the “Severe OSA” group showed lower high-frequency power in normalized units (HFnu) and higher low-frequency power in normalized units (LFnu). The standard deviation of normal R–R intervals (SDNN) and the root mean square of successive R–R interval differences (RMSSD) were independently associated with sleep-disordered breathing. Our findings suggest altered cardiac autonomic regulation with a reduced parasympathetic component in OSA patients and suggest a role of nighttime HRV in the characterization and identification of sleep breathing disorders.

## 1. Introduction

Obstructive sleep apnea (OSA) is a common chronic condition characterized by recurrent upper airway obstructions, resulting in oxygen desaturation and sleep fragmentation [1]. The prevalence of this disorder, which has an enormous societal and economic burden [2], changes by country and region; nearly 1 billion adults aged 30–69 years worldwide were estimated to have OSA [3]. Although easy to manage, this syndrome, which has long been proposed as an independent risk factor for all-cause mortality [4], can adversely affect the patient’s quality of life [5]. The pathological consequences of OSA, which include excessive daytime sleepiness and chronic fatigue, can indeed have dramatic deleterious effects on the work, family, and social life of patients [6]. For example, the disruption of vigilance that can be due to this syndrome has been linked to an increased risk of occupational and car accidents [7].

Importantly, this sleep-related breathing disorder leads to mechanical, inflammatory, and autonomic changes that, in turn, can have potentially serious long-term effects on the heart, circulation, and metabolism [8,9].

OSA-induced autonomic changes appear to play a pivotal role in this context. Oxygen desaturation and hypercapnia induce chemoreflex responses and, thus, sympathetic activation. However, baroreceptive responses to the drop in blood pressure and the lack of pulmonary stretch receptor afferents make sympathetic tone exaggerated during repetitive apneic episodes [10]. Noteworthy, this enhanced sympathetic tone persists during wakefulness and results in impaired cardiac autonomic control, as suggested by heart rate variability (HRV) investigations in OSA patients [11,12]. This is in good agreement with the changes in thickness and/or volume of the cingulate cortex, insula, and thalamus in subjects with moderate-to-severe OSA, revealing a long-term impact of sleep apnea on key structures of the central autonomic network [13]. Therefore, as a measure of cardiac autonomic function, HRV has long been proposed to be a potential marker of sleep-disordered breathing [14,15].

In this study, we measured nocturnal HRV on the electrocardiogram (ECG) tracing of OSA patients as an indicator of the autonomic regulation of cardiac function and tested the utility of nocturnal HRV parameters as predictors of sleep-disordered breathing.

## 2. Materials and Methods

### 2.1. Data Set

The present investigation used the “Apnea-ECG database” [16], which is available via “PhysioNet” [17]. This database consists of 70 nighttime recordings that lasted from ~7 to ~10 h. ECG signals were recorded from 32 subjects and digitized at 100 Hz. Therefore, more than one recording for each subject is included in the original database, which is divided into a “learning set” and a “test set” of the same size (No. = 35). Machine-generated QRS annotations were supplied. Every minute of each recording in the “learning set” was classified as normal or disordered breathing due to the presence or absence of apnea/hypopnea episodes at that time. Specifically, the scoring does not differentiate between apnea and hypopnea events, which were originally counted according to the recommendation of the American Academy of Sleep Task Force [18]. Based on the apnea–hypopnea index (AHI), recordings of the “learning set” fall into one of the following categories: “Severe OSA” (AHI ≥ 30, No. 17 (49%)); “Moderate-Mild OSA” (5 ≥ AHI < 30, No. 6 (17%)); and “Normal” (AHI < 5, No. 12 (34%)). The “learning set” was used to build our predictive model that was then validated on the “test set”. This latter set of data, similar to the learning set (*χ*^2^ _(2, No. = 70)_ = 1.33, *p* > 0.05), includes “Severe OSA” (AHI ≥ 30, No. 14 (40.0%)); “Moderate-Mild OSA” (5 ≥ AHI < 30, No. 10 (29.0%)); and “Normal” (AHI < 5, No. 11 (31.0%)) recordings. The available demographic characteristics of the sample are displayed in Supplementary Tables S1 and S2 for the “learning” and “test” set, respectively. Specifically, the distribution of ECG recordings by sex (learning set: 30 (85.7%) ECGs from male subjects and 5 (14.3%) ECGs from female subjects; test set: 27 (77.1%) ECGs from male subjects and 8 (22.9%) ECGs from female subjects; χ^2^ _(1, No. = 70)_ = 0.85, *p* > 0.05) as well as by body mass index (BMI) (learning set: 11 (31.4%) ECGs from obese subjects, 14 (40.0%) ECGs from overweight subjects, and 10 (28.6%) ECGs from subjects with normal weight; test set: 10 (28.6%) ECGs from obese subjects, 13 (37.1%) ECGs from overweight subjects, and 12 (34.3%) ECGs from subjects with normal weight; χ^2^ _(2, No. = 70)_ = 0.27, *p* > 0.05) did not differ between the learning and test set of data. However, if one analyzes the distribution of ECG recordings by AHI categories and within each set of data separately, a difference between males and females could be observed for both sets of data (learning set, males: 17 (56.7%) ECGs from subjects with “Severe OSA”, 6 (20.0%) ECGs from subjects with “Moderate-Mild OSA”, and 7 (23.3%) ECGs from “Normal” subjects; learning set, females: 0 (0.0%) ECGs from subjects with “Severe OSA”, 0 (0.0%) ECGs from subjects with “Moderate-Mild OSA”, and 5 (100.0%) ECGs from “Normal” subjects; Fisher’s exact test, *p* < 0.01) (test set, males: 12 (44.4%) ECGs from subjects with “Severe OSA”, 10 (37.1%) ECGs from subjects with “Moderate-Mild OSA”, and 5 (18.5%) ECGs from “Normal” subjects; test set, females: 2 (25.0%) ECGs from subjects with “Severe OSA”, 0 (0.0%) ECGs from subjects with “Moderate-Mild OSA”, and 6 (75.0%) ECGs from “Normal” subjects; Fisher’s exact test, *p* < 0.05). Further information and a more thorough description of how the original database was created were reported by Penzel and colleagues [16].

### 2.2. HRV Analysis

HRV measurements were performed as follows. In order to compare the nocturnal HRV parameters between groups, we blindly (i.e., without any information on polysomnography except for the ECG tracing) selected the first 5 min of each hour for the recordings of the three study groups. Therefore, HR and HRV indices were calculated for each 5-min epoch. The HR and HRV data were then averaged for each recording and further as group mean values.

Since the machine-generated QRS annotations were unaudited, R waves were detected through visual inspection of row ECG signals. Ectopic beats and recording artifacts were excluded from the analysis. Additionally, recordings with recognizable cardiac arrhythmias were excluded. Time- and frequency-domain parameters of short-term HRV were quantified based on the 5-min sequence of data [19] using the ChartPro 5.0 software (ADInstruments, Sydney, NS, Australia). In the time domain, the following HRV indices were evaluated: (i) the standard deviation of normal R–R intervals (SDNN, milliseconds (ms)), which reflects all the cyclic components responsible for variability; and (ii) the root mean square of successive R–R interval differences (RMSSD, ms), reflecting high-frequency variations of the R–R interval as an estimate of the parasympathetic input to the heart [20].

A fast Fourier transform (FFT)-based method (Welch’s periodogram: 256 points, 50% overlap, and Hamming window) was applied to obtain the power spectrum. Then, we quantified three frequency-domain parameters: (i) the total power (TP, ms^2^) of the spectrum, which reflects all the cyclic components of variability and is equal to the total variance of R–R intervals, i.e., SDNN squared; (ii) the power of the low-frequency band (LF, ms^2^, 0.04–0.15 Hz), which reflects a complex combination of sympathetic and vagal influences; and (iii) the power of the high-frequency band (HF, ms^2^, 0.15–0.4 Hz), which reflects parasympathetic activity and is related to respiratory sinus arrhythmia. In addition, we calculated the HF and LF power values in normalized units (HFnu and LFnu) [21].

### 2.3. Statistical Analysis

Parametric and nonparametric summary measures of central tendency and dispersion were used as appropriate. We explored the between-variance of nocturnal HRV parameters over the three OSA categories (Severe OSA, Moderate-Mild OSA, and Normal) with one-way ANOVAs. Follow-up analyses were conducted using Student’s *t* tests with a Bonferroni correction for multiple comparisons and Cohen’s *d* as a measure of the effect size. Welch’s *F* followed by Games–Howell post hoc tests was calculated when the homogeneity of variances was not met. The Pearson’s chi-square (χ^2^) test or the Freeman–Halton extension of the Fisher exact probability test was used for the analysis of categorical data and to determine if there was a significant association between nominal variables. Lastly, a polytomous logistic regression model (backward stepwise) was performed to identify the HRV indices capable of predicting the OSA categories. The covariates retained in the final model were SDNN and RMSSD; results were reported as odds ratios (ORs) with 95% confidence intervals (CI). Pseudo-*r*^2^ of Cox–Snell and Nagelkerke as approximate measures of the goodness of fit were also reported. The classification performance of the model was then assessed on the “test set”. Statistical significance was set at *p* < 0.05. All statistical analyses were performed using the software platform SPSS (Statistical Package for the Social Sciences, IBM software package, version 26).

## 3. Results

### 3.1. Nocturnal HRV in Patients with Sleep Breathing Disorders

One-way ANOVA showed that LFnu (*F*_2,30_ = 5.67, *p* = 0.01, ω^2^ = 0.22) and HFnu (*F*_2,30_ = 5.44, *p* = 0.01, ω^2^ = 0.21) varied by study group. Compared to the “Normal” group, the “Severe OSA” group showed lower HFnu (*M* = 21.86 ± 2.46 vs. *M* = 35.54 ± 3.37, *p* < 0.05, *d* = 1.32) and higher LFnu (*M* = 74.43 ± 2.41 vs. *M* = 60.74 ± 3.43, *p* < 0.05, *d* = −1.33) values. However, the sleeping HRV indices did not vary between the “Normal” and “Moderate-Mild OSA” groups nor between the “Severe OSA” and “Moderate-Mild OSA” groups.

### 3.2. Polytomous Logistic Regression Analysis

Table 1 shows the results of the polytomous logistic regression analysis. SDNN and RMSSD values were independently associated with the diagnosis of sleep-disordered breathing. Specifically, as indicated by the final model (top half of the Table 1, left side), SDNN (*b* = 0.10, Wald *χ*^2^_(1)_ = 5.55, *p* < 0.05) and RMSSD (*b* = −0.15, Wald *χ*^2^_(1)_ = 6.76, *p* < 0.01) values significantly predicted whether the subject suffered from severe OSA or not. The OR suggested that a unit increase in SDNN was associated with an 11% increase in the odds of having severe OSA. Conversely, a unit increase in the RMSSD value was associated with a 14% decrease in the odds of having a severe OSA. This model reached an accuracy of 63.6% and classified correctly 88.2%, 50%, and 16.7% of Severe OSA, Normal, and Moderate-Mild OSA subjects, respectively. The “test set” was thus used to validate the model performance. Specifically, our model reached an accuracy of 58.8% and classified correctly 100%, 60%, and 0% of Severe OSA, Normal, and Moderate-Mild OSA subjects, respectively.

## 4. Discussion

Our findings indicate altered cardiac autonomic regulation in OSA patients during nighttime and suggest a predictive role of nocturnal HRV indices in the diagnosis of sleep breathing disorders.

Importantly, impaired cardiac autonomic control has been suggested by several HRV investigations in adult and pediatric OSA patients [8,22,23,24]. For instance, longitudinal analysis of 24 h Holter recordings revealed a reduction in HRV and a shift towards a relative sympathetic dominance in adult OSA patients [12]. Similarly, a decrease in overall HRV and less complex HRV measures during wakefulness have been reported among patients with severe sleep apnea [14], revealing an inverse relationship between HRV and OSA severity. Notably, the same relationship has also been shown in young and healthy adults with subclinical sleep-related breathing disorders [25]. Nevertheless, it is still uncertain what nocturnal HRV parameter might help to discriminate between patients with OSA and healthy subjects. For example, Zhu and colleagues highlighted that, among the classic time- and frequency-domain indices, only the mean normal-to-normal R–R interval could discriminate between these two groups [26]. On the contrary, many authors reported that different time- and frequency-domain HRV parameters could reveal nocturnal impairment of cardiac autonomic function [27,28,29,30]. Their potential use to monitor the impact of OSA treatment on the cardiac sympathovagal balance was also suggested [15,31,32,33,34]. However, the majority of the studies attempting to evaluate the state of sympathovagal balance in OSA patients speculated on very controversial HRV measures (i.e., LF/HF and LF) whose interpretation has been extensively challenged. Therefore, substantial care must be taken when considering HRV investigations in OSA patients. In the present study, lower HFnu and higher LFnu were reported in patients with severe OSA compared to their normal counterparts. Since LF does not solely reflect cardiac sympathetic drive [35,36], these results cannot give any specific information about the sympathetic component. On the contrary, the reduced nocturnal HF power could suggest an overall reduced parasympathetic activity in subjects with severe OSA at night. Taken together, our findings suggest altered cardiac autonomic control in OSA patients but cannot definitely confirm a sympathetic overdrive. On the other hand, despite the methodological heterogeneity (e.g., inclusion criteria, modality of analysis and indices calculated, etc.) that arises when several HRV studies are reviewed, our findings are not necessarily at odds with previous reports that suggest sympathetic overactivity in OSA patients. In this respect, a recent systematic review of 12 studies indicated that adults with OSA displayed a sympathovagal imbalance characterized by higher sympathetic responsiveness and reduced parasympathetic tone [11]. Based on the reduced parasympathetic influence during nighttime, patients with severe OSA might get the beneficial effects of enhanced vagal tone. This is consistent with a diurnal increase in vagally mediated HRV that has been observed in this clinical population following continuous positive airway pressure (CPAP) therapy [37]. Moreover, a recent meta-analysis has suggested that the treatment with CPAP may lead to beneficial cardiac and hemodynamic effects by improving parasympathetic tone in OSA patients and also during sleep [38]. However, an acute and exaggerated increase in parasympathetic activity during sleep could lead to bradycardia and trigger apneas. For instance, vagus nerve stimulation, a coadjuvant treatment for epilepsy, is known to affect sleep breathing and exacerbate the occurrence of apnea/hypopnea events [39,40]. We did not detect significant differences in the HRV parameters between the “Severe OSA” and “Moderate-Mild OSA” groups or between the “Moderate-Mild OSA” and “Normal” groups. This would contrast with the hypothesis that HRV correlates with OSA severity also in the early and subclinical stages of the disease [25]. However, it might also be a consequence of the limited number of recordings available and the resulting poor statistical power. We recommend caution in interpreting our findings.

Finally, the results of our logistic regression analysis suggest that nocturnal HRV can be helpful in the identification of obstructive sleep breathing disorders. Interestingly, SDNN and RMSSD values predicted whether the subject had severe apnea or showed less than five apnea/hypopnea events per hour of sleep. Altogether, our model reached an accuracy of 63.6%, which dropped to 58.8% after validation on the test set. Although this could indicate a poor classification performance, our logistic regression model offers a clear improvement in accuracy compared to the baseline model (i.e., the simplest model that predicts every observation of the test set as belonging to the “Severe OSA” group) which had an accuracy of 41.1%. Regardless, our findings reinforce the usefulness of HRV analysis as an additional tool for OSA identification and characterization [41], highlighting a parasympathetic tone reduction that may not necessarily lead to a reduced nocturnal HRV in patients with sleep breathing disorders.

This work has some limitations. As previously stated, we conducted our research on freely available ECG tracings via the “PhysioNet” database. While this allows full reproducibility of our results, it also limited our investigation to a small number of available recordings. More importantly, we were unable to check for the influence of possible confounding variables, such as sleep phases, lifestyle behaviors, and some clinical features of the sample patients. However, the recordings in the Apnea-ECG database were gathered in baseline conditions, and, therefore, the patients were not under treatment at the time of recording. The patients were recruited because of suspected sleep apnea and hypertension or because of sleep apnea and daytime sleepiness. Antidepressants and psychotropic treatments were excluded. However, the lack of verified data about additional pharmacological treatments known to affect HRV, such as chronotropic drugs (that should be absent at the time of recruitment), prevents drawing firm conclusions. After all, the purpose of this database is to test algorithms and automated ECG-based methods of sleep apnea detection [42,43,44] rather than characterizing HRV dynamics in OSA patients. Since its creation, the Apnea-ECG database, originally built for an engineering challenge, has been widely used and explored [45,46,47]. It makes this database a public source of data with high evidential value. Therefore, to test whether analysis of only 5 min of ECG recording per nighttime hour would be useful in this context, we used a simple approach that, to the best of our knowledge, has never been performed before, although it may seem classical or basic.

Furthermore, severe OSA can lead to important changes in breathing patterns, which could be able to modify HRV rhythmical oscillations per se [48]. Therefore, a direct evaluation of the cardiorespiratory interaction might be required to unveil the nonlinearity and complexity of the coupling patterns in cardiovascular and respiratory systems [49,50]. On the other hand, although only one investigator analyzed the data and it might introduce bias somehow, one strength of the present study is that we visually inspected the ECG tracings rather than using an automated QRS detector or the PhysioNet machine-generated annotations file to ensure correct QRS identification. Moreover, we did not consider controversial HRV indices such as LF/HF [35] in our physiological data interpretation.

In summary, establishing which HRV indices could be more helpful in OSA identification and characterization requires further investigations considering the limitation of HRV application to sleep studies (e.g., the occurrence of repetitive arousals), especially in OSA [48], and it also requires use of integrative approaches, such as different putative markers of this sleep-related condition. However, despite the small-to-medium effect size, our results confirm that altered autonomic modulation of cardiac function in OSA patients during sleep suggests a possible role of nighttime HRV in the identification of sleep breathing disorders. Our findings also draw attention to parasympathetic activity, which might have important implications in assessing cardiovascular risk in patients with OSA. Autonomic changes are known to be one of the most important mechanisms underlying OSA-associated cardiovascular disorders. Importantly, sympathovagal imbalance can be often associated with sleep-disordered breathing even when other related cardiovascular symptoms have not yet appeared. Therefore, even though the association between OSA and cardiovascular dysfunction appears to have a multifactorial basis, autonomic dysfunction might be an early marker of OSA-related cardiovascular sequelae, and investigating nocturnal HRV seems to be a promising and cost-effective screening tool in patients with suspected OSA. Nevertheless, longitudinal investigations are needed to assess how autonomic changes can worsen the cardiovascular consequences of OSA. Moreover, it must be taken into account that, in the present study, we used a database where most recordings come from male subjects and the few coming from females are not equally representative of the investigated AHI categories (i.e., learning set, females: 0 recordings from subjects with Severe OSA, 0 recordings from subjects with Moderate-Mild OSA, and 5 recordings from Normal subjects; test set, females: 2 recordings from subjects with Severe OSA, 0 recordings from subjects with Moderate-Mild OSA, and 6 recordings from Normal subjects). In simple terms, this database is not balanced by sex. This is understandable since men are more likely than women to be diagnosed with sleep-related breathing disorders [51,52]. On the other hand, women seem to show different clinical features of OSA onset (e.g., longer partial airway obstruction, insomnia, etc.) associated with lower AHI scores compared to men. As a consequence, this might lead to the underdiagnosis of OSA in female subjects [53]. Apart from this line of reasoning, the different distribution of AHI categories by sex in the explored database necessitates a careful interpretation of the results and prevents us from extending our findings to the female condition. Additionally, since HRV analysis has currently become a popular tool to investigate cardiac vagal modulation in both animal research and human studies [20,54,55,56,57], the need to address the issue of sex differences in cardiac autonomic function has drawn increasing attention over the past decade [58,59], highlighting a greater resting vagally mediated HRV in women. To further our research, we are thus planning to build up our own database by collecting a similar number of ECG recordings from male and female OSA subjects.

## 5. Conclusions

In the present study, we aimed to evaluate cardiac autonomic regulation in patients with obstructive sleep apnea during nighttime by taking advantage of the Physionet Apnea-ECG Database. As expected, we found that obstructive sleep breathing disorders can be accompanied by changes in cardiac autonomic regulation. Specifically, our findings suggest an altered cardiac vagal function during sleep at night. This might also be associated with an overall sympathetic hyperactivity that has long been speculated for this clinical population. Furthermore, this work shows that some classical indices that describe the nocturnal HRV do predict severe OSA. In particular, we found the most commonly used time-domain parameters of HRV, i.e., SDNN and RMSSD, to sufficiently distinguish between the subjects who had severe apnea and those who showed fewer than five apnea or hypopnea events per hour of sleep. However, these indices did not discriminate the subjects with Moderate-Mild OSA, who are the majority of patients evaluated in the sleep laboratory and could represent an alarming borderline condition. Therefore, this work shows that classical time-domain parameters of HRV could help in discerning whether a subject suffers from Severe OSA or not and provide important information about cardiac autonomic regulation of these patients, even when calculated on brief time intervals via a straightforward analysis. Analyzing only 5 min of ECG recording per night hour and for a few days might be a feasible way to easily obtain enough preliminary information that could also be interpretable by the subject/patient himself and, at least initially, without highly specialized assistance of clinicians. This point might support the development of non-invasive and HRV-based smart monitoring devices. However, the clinical relevance of assessing cardiac autonomic function in patients with suspected OSA based on HRV indices alone may still have limited clinical significance. On the other hand, in this context, HRV analysis might be a time- and cost-effective tool to suggest possible cardiovascular sequelae and provide a solid basis for developing predictive algorithms. According to our point of view, in the next few years, advances in artificial intelligence and the implementation of integrative approaches that should consider different markers of OSA (i.e., inflammatory and metabolic markers, reactive oxygen species, etc.) will lead to dramatic improvements in this field.

## Figures and Tables

**Table 1:** Multinomial logistic regression models (backward stepwise) with “Normal” as the reference category.

	Model 1—Full Model			Model 2			Model 3			Model 4—Final Model		
	95% CI for Odds Ratio			95% CI for Odds Ratio			95% CI for Odds Ratio			95% CI for Odds Ratio		
	Lower	Odds Ratio	Upper	Lower	Odds Ratio	Upper	Lower	Odds Ratio	Upper	Lower	Odds Ratio	Upper
**Severe OSA vs. Normal**												
**SDNN**	0.92	1.06	1.21	0.93	1.05	1.19	0.99	1.09	1.20	1.02	1.11 *	1.21
**RMSSD**	0.77	0.89	1.01	0.77	0.88	1.01	0.78	0.89	1.02	0.77	0.86 *	0.96
**Total Power**	1.00	1.00	1.00	1.00	1.00	1.00	-	-	-	-	-	-
**HFnu**	0.50	0.95	1.82	0.86	0.96	1.08	0.86	0.96	1.07	-	-	-
**LFnu**	0.52	0.99	1.88	-	-	-	-	-	-	-	-	-
**Moderate-Mild OSA vs. Normal**												
**SDNN**	0.83	1.02	1.27	0.84	1.02	1.23	0.89	1.01	1.13	0.90	0.99	1.10
**RMSSD**	0.79	0.95	1.14	0.80	0.95	1.14	0.81	0.95	1.13	0.87	0.98	1.10
**Total Power**	1.00	1.00	1.00	1.00	1.00	1.00	-	-	-	-	-	-
**HFnu**	0.51	0.98	1.90	0.89	1.02	1.16	0.90	1.02	1.16	-	-	-
**LFnu**	0.50	0.96	1.84	-	-	-	-	-	-	-	-	-

Note. Full model: *r^2^* = 0.41 (Cox–Snell), 0.47 (Nagelkerke); *χ^2^* (10) = 17.14, *p* = 0.07. Model 2: *r^2^* = 0.41 (Cox–Snell), 0.47 (Nagelkerke); *χ^2^* (8) = 17.13, *p* < 0.05. Model 3: *r^2^* = 0.40 (Cox–Snell), 0.46 (Nagelkerke); *χ^2^* (6) = 16.63, *p* < 0.05. Final model: *r^2^* = 0.37 (Cox–Snell), 0.43 (Nagelkerke); *χ^2^* (4) = 15.45, *p* < 0.01. * = *p* < 0.01.

## Data Availability

This study was performed on ECG tracings from Apnea-ECG Database, which is freely available via “PhysioNet.org” (accessed on 1 February 2023). All relevant data are included in the manuscript and supporting information are available from the authors upon request.

## Supplementary Materials

The following supporting information can be downloaded at: https://www.mdpi.com/article/10.3390/biology12040533/s1, Table S1: Learning set characteristics; Table S2: Test set characteristics.
