# Supplementary material for: Nocturnal Heart Rate Variability Might Help in Predicting Severe Obstructive Sleep-Disordered Breathing
Source: Biology (Basel). 2023 Mar 31;12(4):533. doi: 10.3390/biology12040533 (PMC10135696; doi:10.3390/biology12040533)
Supplement: Supplementary file 1 [file biology-12-00533-s001.zip › biology-2249742-supplementary.pdf]

Supplementary Table S1. Learning set characteristics. Please, note that the demographic characteristics are shown for each recording.

Recording	Sex	Age	AHI (events/h)	Height (cm)	Weight (Kg)	BMI (category)
**L01**	Male	51	69.6	175	102	33.31 (O)
**L02**	Male	38	69.5	180	120	37.04 (O)
**L03**	Male	54	39.1	168	80	28.34 (OW)
**L04**	Male	52	77.4	173	121	40.43 (O)
**L05**	Male	58	41	176	78	25.18 (OW)
**L06**	Male	63	24.7	179	104	32.46 (O)
**L07**	Male	44	63	177	105	33.52 (O)
**L08**	Male	51	42	179	88	27.46 (OW)
**L09**	Male	52	31.7	178	82	25.88 (OW)
**L10**	Male	58	21	176	78	25.18 (OW)
**L11**	Male	58	14	168	103	36.49 (O)
**L12**	Male	52	80.2	173	121	40.43 (O)
**L13**	Male	51	42	179	88	27.46 (OW)
**L14**	Male	51	54.7	175	102	33.31 (O)
**L15**	Male	60	52	176	113	36.48 (O)
**L16**	Male	44	41	177	105	33.52 (O)
**L17**	Male	40	33	179	96	29.96 (OW)
**L18**	Male	52	82.4	178	82	25.88 (OW)
**L19**	Male	55	34	178	90	28.41 (OW)
**L20**	Male	58	41	176	78	25.18 (OW)
**L21**	Female	44	0.24	170	63	21.80 (NW)
**L22**	Male	53	19	176	85	27.44 (OW)
**L23**	Male	53	24	176	85	27.44 (OW)
**L24**	Male	42	0.7	180	64	19.75 (NW)
**L25**	Male	52	5	180	135	41.67 (O)
**L26**	Male	31	0	184	74	21.86 (NW)
**L27**	Male	37	0	180	83	25.62 (OW)
**L28**	Male	39	0	184	65	19.20 (NW)
**L29**	Female	41	0	180	65	20.06 (NW)
**L30**	Female	28	0	169	57	19.96 (NW)
**L31**	Female	28	0.25	171	65	22.23 (NW)
**L32**	Female	30	0	168	56	19.84 (NW)
**L33**	Male	42	0	180	64	19.75 (NW)
**L34**	Male	37	0	180	83	25.62 (OW)
**L35**	Male	27	0	184	72	21.27 (NW)

AHI, Apnea-Hypopnea Index; BMI, Body Mass Index; NW, normal weight; O, obese; OW, overweight.

Supplementary Table S2. Test set characteristics. Please, note that the demographic characteristics are shown for each recording.

Recording	Sex	Age	AHI (events/h)	Height (cm)	Weight (Kg)	BMI (category)
**T01**	Male	44	63	177	105	33.52 (O)
**T02**	Male	46	37.7	167	69	24.74 (NW)
**T03**	Female	44	0.13	170	63	21.80 (NW)
**T04**	Male	39	0	184	65	19.20 (NW)
**T05**	Male	55	34	178	90	28.41 (OW)
**T06**	Male	31	0	170	66	22.84 (NW)
**T07**	Male	58	21	176	78	25.18 (OW)
**T08**	Male	55	48	178	90	28.41 (OW)
**T09**	Male	43	18.5	177	80	25.54 (OW)
**T10**	Male	39	10	170	131	45.33 (O)
**T11**	Male	52	5	180	135	41.67 (O)
**T12**	Male	40	33	179	96	29.96 (OW)
**T13**	Male	57	18.7	171	97	33.17 (O)
**T14**	Male	38	79.5	180	120	37.04 (O)
**T15**	Male	63	15.9	179	104	32.46 (O)
**T16**	Male	53	24	176	85	27.44 (OW)
**T17**	Female	27	0	158	53	21.23 (NW)
**T18**	Male	27	0	184	72	21.27 (NW)
**T19**	Male	54	56.2	168	80	28.34 (OW)
**T20**	Male	51	43	179	88	27.46 (OW)
**T21**	Male	53	19	176	85	27.44 (OW)
**T22**	Female	27	0	158	53	21.23 (NW)
**T23**	Male	43	14.3	177	80	25.54 (OW)
**T24**	Male	31	0	170	66	22.84 (NW)
**T25**	Male	55	48	178	90	28.41 (OW)
**T26**	Male	57	15.1	171	97	33.17 (O)
**T27**	Male	60	75	176	113	36.48 (O)
**T28**	Male	60	75	176	113	36.48 (O)
**T29**	Female	41	0	180	65	20.06 (NW)
**T30**	Male	44	41	177	105	33.52 (O)
**T31**	Female	29	93.5	183	100	29.86 (OW)
**T32**	Female	29	71.8	183	100	29.86 (OW)
**T33**	Female	28	0.13	169	57	19.96 (NW)
**T34**	Female	30	0.38	168	56	19.84 (NW)
**T35**	Male	31	0	184	74	21.86 (NW)

AHI, Apnea-Hypopnea Index; BMI, Body Mass Index; NW, normal weight; O, obese; OW, overweight.
